# Embedded self-conceptualization and social learning in online social networking platforms

**DOI:** 10.3389/fpsyg.2022.901625

**Published:** 2022-08-30

**Authors:** Yan Yu

**Affiliations:** School of Information, Renmin University of China, Beijing, China

**Keywords:** social networking, learning outcomes, e-learning, social learning theory, self-concept

## Abstract

Online social networking (OSN) has deeply penetrated university campuses, influencing multiple aspects of student life. Standing from a pedagogical perspective, this study investigates how university students’ OSN engagement affect their learning outcomes. Drawn upon social learning theory, this study proposes that OSN engagement help university students’ establishing the self-efficacy belief, achieving social acceptance and acculturation with environment, and these attributions further lead them to attain positive learning outcomes which are shaped by self-esteem development, satisfaction with university life, and the grade point average (GPA) based performance. Results from a survey accompanied by focus group discussions support these embedded self-conceptualization and social learning in OSN. This study contributes to the extant research on OSN for learning by highlighting the role of OSN for the whole person development, especially the students’ self-conceptulization and psychological well-beings. The revealed mediating mechanisms also adds values to social learning theory and imply the design foci of e-learning activities and applications.

## Introduction

Online social networking (OSN) creates new types of communication, collaboration, and interactional activities for interpersonal relationships, which has deeply penetrated people’s lifestyle and thus raises the awareness of socially and pedagogically innovative trajectories. OSN be regarded as one educational technology when it is used to engage learners in appropriate learning activities. It would be an inadequate usage of modern technology if it is simply to be treated as a transmission medium. The real value of technology for education is that it can be used to engage students in appropriate learning activities and thus leads them to achieve certain learning outcomes.

In social networking platforms (e.g., Facebook, Twitter, Linked-in, Instagram, etc.), people, including university students, are amazed by their rapidly snowballed social networks and the rich learning information and content. “Typing oneself into profile” caters to the needs of self-expression and self-identity development (Kreps, 2010), and virtual relationships make individuals capable to gain diversified information (Wasko and Faraj, 2005), both of which mirror one’s active engagement in using new technologies for social networking and learning. In such a networked meta world, those activities of self-expression and interactions shape new learning opportunities and learning experiences in online networks and communities (Feyzi Behnagh and Yasrebi, 2020; Manca, 2020; Ouyang et al., 2021). Meanwhile, prior studies explored the relationship between OSN and academic performance and found both negative and positive effects (Junco, 2012; Paul et al., 2012; Troussas et al., 2020, 2021). Nevertheless, learning outcomes are multifaceted. Therefore, this paper focuses on the primary question: how does OSN lead university students to achieve learning outcomes.

The answers to the above question directly determine what pedagogical implications OSN can derive for universities and individual students. Without a deep investigation, universities might not know whether it is appropriate for them to engage students to learn through OSN and in what way the learning practices could be designed, although they may have already recognized that OSN is influencing and changing the university students’ learning behavior (Liu et al., 2017).

The “whole person” development goal of general education in most universities offers a niche space for OSN to contribute to education. “Whole person” development requires universities to not just concentrate on students’ academic performance but also provide learning opportunities for developing their self-recognition, identity, healthy morality and affection. These examples of psychological well-being cannot be realized by typical in-class curricula but are cultivated in student social life. The rapid changing of social media and networking technologies are altering educational systems. Social networking has constituted an important aspect of university students’ learning regarding university itself is a social system of individuals’ interacting within a shared academic context. In a platform-enabled learning context, university students tend to self-regulate their learning and interact with peers for feedback, and engage in OSN for physical and psychological well-being (Yu et al., 2010a,b; Lambropoulos et al., 2012; Dooley, 2020; Ouyang et al., 2021). Therefore, this study aims to develop a systematic framework for elaborating how university students’ OSN engagement bring them multifaceted learning outcomes.

This study relies on the constructivism-based learning theories to develop a comprehensive research framework. Piaget’s (1953) notion of active agency highlights the individual engagement in learning activities. Further, Bandura (1977) social learning theory, which is the main theoretical base of this research, provides a systemic framework for the investigation of individuals’ learning and cognitive development in a social context. This theory points to the significance of the individuals’ self-regulation, interactions with peers and the external situation on one’s learning. Other related theories and literature (Vygotsky, 1978; Alavi, 1994) have also emphasized the salience of these interactions for learning. Individuals often self-initiate and regulate their learning to achieve desirable learning outcomes, including improved cognition, affection, and skills.

Given self-regulation capacity, students generate creative learning activities via OSN. They disclose their opinions online to attract others’ attention and expand their network scope through which they receive diversified information and support. Through these activities, university students experience self-recognition, disclosure, reciprocal appreciation, and mutual knowledge construction, thus shaping their socialization to the university society in which they establish efficacy beliefs, gain social acceptance from others, and learn university culture. Consequently, they can better articulate their role, attain better performance, and develop commitment to the university. These aspects are important for students to develop as a “whole person,” acquiring both skills and psychological well-being that are conducive to lifelong learning.

Research on the learning impacts of OSN on university students has important pedagogical implications for both individual learning and educational administration. From the individual perspective, it has been found that “net generation” learners have different styles of information processing and learning expectations. This empirical research is expected to inform individual students of the impact that their engagement in online social networks can have on them. From the institutional perspective, it is also necessary for universities to be aware of the learning impacts of OSN that has been ubiquitous among students and has affected their behavior, since most universities increasingly emphasize student-centered learning practices that have a deep theoretical root in constructivism. The social networking based e-learning tools and related algorithms are developed (Troussas et al., 2015, 2020, 2021). The new technology-driven behavioral changes behoove educational institutions to consider engaging students into OSN for learning and thus reconsider traditional pedagogical approaches. As such, this study contributes to the extant research by highlighting the role of OSN for the whole person development and by revealing the mediating mechanisms from OSN engagement to learning outcomes.

## Literature review and theoretical background

### Learning outcomes

Learning outcomes are constituted by three aspects, including the cognitive, affective and skill-based aspects (Kraiger et al., 1993; Schmidt and Ford, 2003; Zhang et al., 2021). The knowledge-based cognitive aspect is similar with intellectual learning that concerns one’s comprehension and application of knowledge about subjects, which is shaped by declarative knowledge and one’s mental model and metacognition. The attitudinal-based affective aspect falls into emotional learning shape by their feelings and ability to interact with situations. Individuals’ attitudes toward and appreciation of their learning experience are important indicators of affective learning outcomes. The skill-based aspect of learning outcomes is about the development of critical thinking and the technical skills to perform tasks. These outcomes are often reflected by their behavioral performance (Leidner and Jarvenpaa, 1995). For individual students, their university life consists of academic and social parts. Depending on the different learning objectives, the above three domains can be divided into two aspects: course/discipline based academic learning and social learning. This study is focused on social learning. University students’ social learning experience is important to their “whole person” development which is not just limited in the knowledge obtained from in-class curriculum but also emphasizes students’ psychological well-beings in a long run. University students’ self-esteem, satisfaction with university life, and grade point average (GPA) based performance can be specified to represent the cognitive, affective, and skill-based domains of learning outcomes, respectively.

Self-esteem is a personal judgment of worthiness expressed in the attitudes that individuals hold (Burns, 1982, p6). This implies that individuals feel worthy, that is, they respect themselves for what they are and do not condemn themselves for what they are or are not. The development of self-esteem in university involves the kind of cognitive building that allows university students to recognize who they are and abilities they have or desire, and thereby value themselves. As an affective outcome of learning, satisfaction with life represents university students’ attitudes about their learning experiences in the university.

GPA-based performance is a summative assessment of students’ knowledge and skills gained while at university. The assessment on students can be formative and summative. The formative assessment is progressive, pursuing for iterative feedback during learning, while the summative assessment is usually conducted in the end of a learning episode, serving as a conclusive outcome of learning. GPA provides an index of how successfully students have learned in university from both academic and social aspects. GPA reflects students’ knowledge level, the ability of problem-solving. Also, it embodies students’ self-regulatory behavior a long-run learning process.

### Learning theories

Learning theories on education are drawn upon two paradigms: phenomenography versus constructivism. Phenomenography, coined by Marton (1981), postulates that teaching is a matter of changing the learner’s perspective and that the goal of learning is to understand and assimilate the reality. In contrast, the constructivism is based on the idea that the learner is an active agency who has to engage in learning to create knowledge (Piaget, 1953).

Further, the social constructivism sheds lights on the social environment in which interaction with the contextual elements (e.g., peers) play a crucial role in one’s learning and creation of new knowledge. Bandura’s (1977) social learning theory Vygotsky’s (1978) developmental psychology in education, and Lave and Wenger’s (1991) practice of community enrich the social constructivism from different perspectives but with fundamental consistency. To fulfill the whole person development goals of modern general education, constructivism-driven teaching and learning is increasingly emphasized by universities.

#### Active agency

The first key concept in constructivist thinking is active agency. People are seen as actively constructing their world of knowledge, of values, of social interaction, or of social and emotional adaptation. Jean Piaget is, perhaps, the most influential theorist on active agency in relation to knowledge construction and cognitive development. According to Piaget (1953), people, from infancy onward, actively and continuously organize and re-organize information and experiences in their physical and virtual worlds for progressive adaption. Essentially, Piaget regards that the active agency adapts to the situated environment ‘from the inside out’.

Piaget’s notion of active agency in cognitive development underscores the necessity of engagement for learner to explore the physical and social world because personal development does not just happen to human agency. Although the inheritance of individual learner (the nature of learning) may determine how well the individual can achieve certain learning outcomes, the chances of experience (nurture learning) constitute another premise of learning. For university, this means providing students with opportunities to “try things out” (Donald et al., 2002).

The notion of active agency is also shaped in the self-regulated learning theory in which students are supposed to be self-regulative (Zimmerman, 1989). Students are seen as metacognitively, motivationally, and behaviorally active learners who tend to use various processes to regulate their learning. In this sense, self-regulation goes beyond the active engagement itself. Self-regulation is more demanded in e-learning context because students are offered with learning autonomy. Thus, it is appropriate to engage students to learn via OSN where social learning activities have emerged among students.

#### Social learning theory

The second key concept in constructivism is that of social context where interactions occurs and learning is nurtured. While Piaget essentially see the learner as actively adapting to the situated environment ‘from the inside out’, social theories (Bandura, 1977; Vygotsky, 1978; Lave and Wenger, 1991) recognize the learner as active, with learning essentially mediated “from the outside in.”

Social learning theory (Bandura, 1977) emphasizes the social context of learning in which the prominent roles of symbolic, vicarious, and self-regulatory processes are assumed in psychological functioning. People are assumed to be anticipative, purposive, and self-evaluating proactive regulators of their motivation and actions (Bandura and Locke, 2003).

• First, people have an extraordinary capacity to use symbols for event representation, experience analysis, as well as communication with others. The symbolic process indicates the intent and capacity of people to express themselves.
• Second, people learn from direct experiences as well as by observations of others’ behavior and the consequences of that behavior. Modeling influences, principally through their informative functions, produces learning by matching the modeled activities to the consequences after which they decide upon their behavior.
• Third, people serve as principle actors of their own change rather than as simple reactors to environmental influences. They can identify, utilize, and transform the stimuli impinging upon them. They can also exercise measures of control over their own behavior by a self-directed and regulated process.
• Fourth, social learning theory argues for a mutual action between personal and environmental attributes for learning. There are continuous reciprocal interactions between people and their situated environments that shape people’s adaptation to the environment.

Two other learning theories are relevant to Bandura’s social learning theory – the developmental theory (Vygotsky, 1978) and the situated learning theory (Lave and Wenger, 1991). Vygotsky’s theory stresses the role of mediator in the cognitive development, i.e., learning occurs through interaction with knowledgeable others. This theory posits that individuals’ social interaction precedes their cognitive development, and individual cognition is a product of their socialization. Vygotsky defines the zone of proximal development (ZPD) in which individuals learn through interaction and scaffolding. This means that individuals learn improved skills under the guidance or in collaboration with more knowledgeable others who can be teachers, coachers, and peers. Although Vygosky emphasizes the mediation by social interaction, his theory is based on such an assumption that a learner is an active agent who can construct knowledge through the processes of social interaction, not just passively receive knowledge.

Lave and Wenger (1991) propose the situated learning model in a community of practice. They conceive of learning in terms of legitimate peripheral participation by which newcomers interact with the situated environment (community), learn the rules, norms or cultures, and establish membership. They can eventually become the core of the community as they reproduce the meaning of the community. Essentially, the legitimate peripheral participation describes one’s engagement in social practice in which learning is embedded. Such participation or engagement shapes the interaction between the learner and the context and facilitates learners to negotiate or renegotiate with the community, thus allowing them to learn more knowledge skills.

To sum up, the above learning theories rooted in constructivism consistently emphasize the pivotal role of the active agency’s engagement, self-regulation and social interaction in learning. Despite fundamental consistency, they can still be differentiated from one another. Bandura’s social learning theory elaborates the salience of self, peers, and situations for achieving learning outcomes, depicting an integral social learning process. As complementary to the social learning theory, the developmental learning theory and the situated learning theory focus on different aspects of social interaction. Vygotsky creates a developmental education psychology and highlights a learner’s interaction with knowledgeable others, whereas the situated learning theory sheds light on the learner’s interaction with the broader environment, context and culture.

## Research model development and hypotheses

### Research framework

**FIGURE 1:**
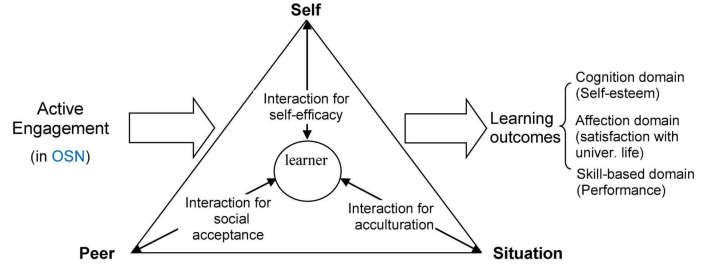
Embedded social learning in an online social networking framework.

According to Bandura’s social learning theory and related theories, individual learners, peers, and situations are three basic elements of social learning. Individual learners will self-initiate learning for expression or representation purposes by engaging in specific activities. Engagement is energy in action, connecting the human agency to the activity. Engagement thus reflects a student’s active involvement in the learning activities. Further, individual learners can self-regulate the learning process by interacting with peers and situations, as well as in personal reflection. These interactions constitute an integral social learning process that leads individual active engagement to desirable learning outcomes in multiple domains, as shown in Figure 1.

More specifically, the above theoretical perspectives suggest that individual engagement enacts social learning processes in which individuals establish self-concepts and self-efficacy beliefs, get social acceptance via vicarious learning (i.e., observe and appreciate others and reciprocally get acceptance from others), and accomplish acculturation to situations (i.e. assimilate the rules, norms and culture of the situated environment and adapt to it). The social learning processes play important roles in transforming individuals’ initial engagement or effort into certain outcomes that are shaped by their self-esteem, satisfaction with university life, and GDP-based performance.

Online social networking shapes one aspect of self-initiated and self-regulated learning behavior. On a social networking platform, individuals design personal profiles for exposure to the public, establish various relationships, update their status, express feelings, join sub-communities, and generate or participate in social activities with others. These engagements categorize participants into being, leading individuals to know themselves, be satisfied with life, and gain skills for performance improvement. Thus, OSN can have a direct impact on their learning outcomes. Further, individual OSN engagement activates the social learning processes composed of self-efficacy beliefs, social acceptance through peer interactions, and acculturation through interactions with the situated environment. These processes play a prominent role in bridging engagement to designated learning outcomes. Engagement emphasizes the time and energy put into the social network websites, in which the underlying socio-psychological processes are embedded and largely influence individuals’ cognition and behavior. Social learning theory underscores these interactions boosted socialization processes for achieving specific learning goals or outcomes. Individuals students’ socialization such as interacting with peers and their situated universities play an important role in affecting their learning outcomes (Yu et al., 2010b; Tian et al., 2011). Hence, the learning impacts of OSN engagement can be interpreted from three aspects.

### Online social networking engagement and learning outcomes

Social learning theory (Bandura, 1977) stresses self-directed learning, suggesting that individuals’ self-initiated active engagement functions as an initial motive for achieving desirable learning outcomes. In most cases, individuals will self-initiate, regulate learning and actively construct knowledge by acquiring, generating, and structuring information. They can use symbols to represent events, analyze their conscious experience, communicate with others, and engage in insightful actions.

Online social networking (OSN) platforms, such as Facebook, Twitter, Linked-in, etc., provide a new approach for university students’ learning. Through OSN, individuals are equipped with an extraordinary capacity to express themselves, establish various relationships, and interact with others at any distance in time and space, thus addressing their self-expressive, networking and informational needs. To activate such learning and fulfill these needs, OSN engagement is required – meaning that time and psychological energy are devoted to these sites.

For self-expressive purposes, individuals present themselves in an online viewable profile and articulate their social networks on the virtual platforms. The profile provides individuals with an opportunity not only to articulate the intended expression but also to receive peer validation. As important identity signals, public and virtually displays of connections help people navigate the networked social world, and this extended network can serve to validate self-conceptualization presented in profiles (Ouyang et al., 2021).

For networking and informational needs, individuals establish and maintain extensive relationships with peers and selectively develop further interactions on these social networking platforms. These advanced social networking applications greatly expand their learning objectives (i.e., connected friends) and their information seeking scope. They have the dual advantage of mimicking the targeted models/peers by viewing the profiles and exploring the hobbies, interests, or specific knowledge of others, and learning more about the university environment by joining a university network, thus finding the information that typifies real life at university. Other than developing a large network of various contacts, individuals spend time to cultivate close relationships with a small group of people who can provide reliable information and genuine social support. Thus, the typical structure of individuals’ OSN is often in a core-periphery mode. According to classic social network research, individuals can gain instrumental value from having a large-ranged network of non-redundant weak ties to attain task-related outcomes (Granovetter, 1973), and they can also receive reliable and consistent knowledge from the smaller, tighter group of strong ties (Coleman, 1990). By OSN engagement, individuals take advantage of both the large spectrum of relationships and the small group of close relationships, both of which can offer different information for learning.

In educational contexts, social networking behavior is related to learning and academic success by self-presentation and the creation of a system of information, contacts and support. Self-presentation by online profile is essentially self-conceptualization. The profile is based on university students’ recognition of who they are and what they are doing or want to do, thus facilitating the establishment of self-esteem. When they engage in OSN platforms, they often form their own communities or groups based on shared interests or other shared characteristics. Such a living learning community is beneficial for students’ affective development and satisfaction with university life, as the information and communication in these communities are likely to help them integrate their social and academic lives in the university. University students with more engagement in social networking can have better mental health and affective development (Steinfield et al., 2008; Sánchez et al., 2014). Furthermore, self-directed OSN offers students intellectual autonomy, enhances their information literacy, and promotes creative communication skills, which are key to their academic success (Kraiger et al., 1993; Hwang et al., 2004). Thus, the above leads to the following hypotheses:

H1: University students’ OSN engagement is positively related to their self-esteem development (H1_*a*_), satisfaction with university life (H1_*b*_), and performance (H1_*c*_).

### Mediation role of social learning processes

The hidden complexity and dynamism of social networking behavior necessitates exploration of the underlying linkage mechanism between students’ OSN engagement and the aroused learning outcomes. According to social learning theory (Bandura, 1977), the information searching and feedback receiving (especially from similar others) in self-initiated and regulated social networking will greatly facilitate individuals’ socialization and adaptation to the situated environment in which they learn to value their own capabilities, interact with peers and become accepted by peers, and learn the university’s culture (Yu et al., 2010a). Thus, university students build their own self-efficacy beliefs, social acceptance and acculturation, which are three important processes that translate students’ initial social networking engagement into their desired outcomes.

#### Role of self-efficacy belief

***Self-efficacy*** refers to personal beliefs about one’s capabilities to learn or perform skills at designated levels (Bandura, 1986). Learners acquire information to appraise their self-efficacy from their performance accomplishments, vicarious (observational) experiences, and forms of persuasion. Despite students’ own performance that offers a reliable guide for generating efficacy beliefs, information from social interaction will also influence students’ perception of their self-efficacy. Schunk (1994) argued that learners acquire efficacy information by socially comparing themselves with similar others. When students observe peers performing a task, they are apt to believe that they also have the capability to accomplish it. Learners often receive valuable persuasive feedback from peers, professors, and family members, leading them to believe that they are capable of performing tasks (e.g., “You can do this”). Noteworthy is that the acquired information does not influence self-efficacy automatically, but rather is cognitively appraised. Thus, the self-efficacy belief could be enhanced by positive information.

OSN platforms provide new channels for individuals to observe others and receive information. University students can establish friendships ranging from intimate relationships to simply being acquainted via OSN platforms. They can review their friends’ activities listed on their own homepages, and then compare and mimic their friends’ actions, especially those of intimate friends, thus attaining efficacy beliefs after accomplishing the achievable activities. Students also receive messages from their intimate friends which often express positive sentiments and encouragements. This feedback helps to relieve the psychological pressure and even academic anxiety of students, thereby developing efficacy beliefs of university life. The hypothesis thus follows that:

H2: University students’ OSN engagement is positively related to their development of self-efficacy belief.

Individuals’ self-efficacy beliefs influence their thoughts, emotional reactions and behavior (Bandura, 1986). People do not seek out or enjoy doing things that they believe they cannot do well. People with high efficacy beliefs are more willing to persist in tasks, reduce fear and anxiety, have positive emotions, focus on problem-solving strategies, and thus achieve a higher level of outcomes (Zimmerman, 1995). As such, individuals self-esteem is internally promoted (Burns, 1982). Self-efficacy beliefs lead individual students to feel confident of their capabilities to perform tasks. This indicates important evaluative information for students to positively appraise themselves, develop self-esteem and be satisfied with life in the university. Further, university students’ self-efficacy beliefs play a positive influential role on their performance. A number of studies have shown the significant contribution of self-efficacy beliefs to individuals’ performance attainment, including academic achievement and work-related performance (Bandura and Locke, 2003; Zhang et al., 2021). Accordingly, the hypotheses follow that:

H3: University students’ self-efficacy belief is positively related to their self-esteem development (H3_*a*_), satisfaction with university life (H3_*b*_), and performance (H3_*c*_).

#### Role of social acceptance

***Social acceptance*** concerns individuals developing satisfying relationships with peers and becoming associated (Bauer et al., 2007). Interacting with peers is an important facet of socialization (Schein, 1968; Chao et al., 1994). The personal profile on Facebook is not a mere static representation of self but a communicative body in conversation with other represented bodies, having the goal of gaining peer validation (Kreps, 2010). Peer interaction on Facebook is also embodied by enacted social activities, e.g., sending and receiving comments, greetings, or gifts, poking and re-poking friends, inviting friends to join a group, and so on. However, meaningful peer interaction usually takes place in a small dense network of strong ties that provide social support and a sense of belonging. In a tightly knit network, individuals are likely to convey reliable and consistent information and social cues with one another that enable their understanding of peers’ interests, expertise, and development of commonality. Such a network that nurtures close peer-interactions allows individuals to learn more about their peers and gain social acceptance from them. Individuals tend to cultivate strong relationships with a relatively small group of people, even though their total friendship scope is expansive. Following the rationale of a close social network view, OSN is expected to promote university socialization. Thus, the hypothesis follows that:

H4: University students’ OSN engagement is positively related to their social acceptance by peers in the university.

Peers play an important role in one’s learning. Vygotsky (1978) emphasizes the impact of others in his developmental learning zone, that is, peers can assist in drawing insights into personal style and self-concept development. According to Burns (1982), self-concept is forged out of the influences exerted on the individual from outside, particularly from people who are the significant others. As social people, individuals also find themselves judged by the criteria of society or relevant subgroups, apart from the criteria of their own making. These “other” people influence how the individuals interpret themselves. Walsh et al. (1998) purport that by “forming mutual and meaningful connections with others, individuals gain a greater sense of energy, purpose, vision and ultimate self-understanding.” Thus, the formation of self-esteem involves the internationalization of society or the related subgroup’s judgment, although it is primarily internally promoted. Being accepted by peers and integrating a self into the connected peers influence the individuals’ sense of esteem and worthiness. Gaining information from others that they are liked and respected produces satisfaction for the individuals’ self-esteem need. Further, peers can provide emotional and psychological support that facilitates individual learning and academic satisfaction. Individuals who are socially accepted by peers may also achieve higher performance because the relationships they form with peers are social capital that potentially facilitates their skill development and performance enhancement (Sanchez et al., 2006; Bauer et al., 2007; Sánchez et al., 2014). Ginsburg-Block et al.’s (2006) meta-analysis also sheds light on peer influences on individuals’ learning, including their self-conceptualization, affection and behavioral learning. The above justifies the following hypotheses:

H5: University students’ social acceptance by peers is positively related to their self-esteem development (H5_*a*_), satisfaction with university life (H5_*b*_) and performance (H5_*c*_).

#### Role of acculturation

***Acculturation*** refers to individuals gaining an understanding of environmental norms and cultures (Schein, 1968; Morrison, 1993). The interaction between individuals and situated environments constitutes the other facet of socialization. In the educational context, acculturation specifically refers to university students’ understanding of the university culture, norms, policies and educational goals. According to Lave and Wenger’s (1991) legitimate peripheral participation, acculturation essentially is a fresh student movement from the periphery to the center of the community of a university by assimilating the environmental cultures and rules and/or creating new meanings for the community.

To complete the process of acculturation, students need to seek normative information about the university (Morrison, 1993), usually done through various channels. Social network research suggests that a network of diverse members greatly facilitates access to useful information, as such diversity enables individuals to tap multiple pockets of information and knowledge, thereby providing more comprehensive views for individuals to understand multi-faceted environments. As Morrison (1993) observes, a large range of networks with broader information is beneficial for individuals’ learning about an environment’s attributes (e.g., norms, policies and culture). University students’ social connectedness affects their learning about the university and the resultant commitment and retention. As aforementioned, OSN enables individuals to expand their network range, thus potentially providing more diverse information access channels to obtain the normative information of the situated university, e.g., by connecting the university alumni, joining the particular university communities, browsing others’ comments on the university, and even by connecting with the President of the university. These learning activities facilitate the interaction between the individual students and the university by which the students experience the acculturation to the university. The above leads to the following hypothesis:

H6: University students’ OSN engagement (on Facebook) is positively related to their acculturation in the university.

A supportive environment is also key to individuals attaining desirable learning outcomes. Social learning theory (Bandura, 1977) states that “the environment is only a potentiality until mobilized by appropriate interactions which cannot inevitably impinge upon individuals.” It is individuals’ interaction with the environment that causes their behavioral consequences. Thus, acculturation shaped by individual students’ understanding and assimilation of the environmental attributes, such as culture, norms, values and goals of the university, can exert influences on the students’ cognition, affection, and skill development.

From the cognitive learning perspective, self-esteem is also influenced by the embedded environment that generates broader values and norms, other than just being internally promoted. The learning of general educational goals and values of universities helps students clarify or reinforce the goals of self-regulated learning embedded in OSN. Given the goodness of the goal claimed by the universities, the students’ buy-in of the university culture, values and goals facilitates their self-concept development and enhances their self-esteem when studying. From the affective learning aspect, acculturation that represents a certain degree of congruence between the value advocated by the university and the internal value believed by students themselves, nurtures and maintains individual students’ integrity and commitment to the university. Such integrity and commitment largely leads students to greater satisfaction with university life, promoting retention (Thomas, 2000). Individual students’ knowledge of situated universities with value congruence will motivate them to make more effort to achieve better performance. The above leads to the following hypotheses:

H7: University students’ acculturation to the university is positively related to their self-esteem development (H7_*a*_), satisfaction with university life (H7_*b*_) and performance (H7_*c*_).

## Research method

This study adopted multiple methods, including focus group discussion and survey, to explore and validate the research model. Survey was chosen as the primary methodological basis to test the hypotheses. Focus group discussion is complementary to the survey approach with respect to the interpretation of relationships and clarification of puzzling findings (Jick, 1979). This methodological triangulation helps to improve the validity of judgments. The adopted qualitative-quantitative method is in line with the spirit of multimethod research (Mingers, 1997).

### Focus group discussion

Before implementing the main survey, four rounds of focus group discussions involving 14 undergraduates in the same university were conducted. The implementation of focus group discussion followed the recommendation by Nyumba et al. (2018). Each round of discussion was composed of three to four university students across cohorts, with discussions lasting about 1 h. These discussions explored the university students’ OSN behavior and their comments on the impact of the OSN of their university life. To elicit students’ attitudes, four questions were designed for discussion: (1) What do you usually do on the existing OSN platforms, e.g., Facebook? (2) What have you learnt from your experience on Facebook? (3) What are your expectations on using Facebook for educational purposes? (4) What is your opinion when OSN is used for college education? The discussions were recorded and transcribed for discourse analysis. Three main themes were identified, including self-expression, skill improvement, and attitudes toward using OSN for education.

During the discussion, the interviewed students expressed their Facebook passion. They logged onto Facebook at least once a day and showed excitement for the number of their online friends, demonstrating that the main motivation of being on Facebook is to build and maintain friendships. On Facebook, they presented their real “self,” expressed feelings to connected friends, and expected to receive friends’ validation. They commented that:

*“Being on Facebook becomes a daily activity and we log on Facebook multiple times per day.”*

*“I am excited of my number of friends, a big network. I have three hundreds of friends. How many you have? [to another interviewee] Two hundred? Mine is higher [smiling].”*

*“I usually use my real name and real photo on Facebook and mainly connect with the recognized friends. I want my friends to know who I really am.”*

*“I like to track my friends’ status and also be tracked. It’s exciting to get my friends’ comments or feedbacks when I express my feelings on Facebook. It’s like a support and I feel very warm.”*

The interviewees agreed that the experiences on Facebook could improve certain skills such as social skill, information literacy, collaboration, and self-esteem. They commented that:

*“Facebook makes me less shy.”*

*“I learnt how to have effective communications. I also learnt some knowledge through blogs of my friends in Facebook.”*

*“I learnt sharing from [using] Facebook. Before, I seldom share my photos nor provide comments to others.”*

*“Facebook is more than Blackboard. On Facebook, I can connect with the lecturers and students in terms of social friendship, not limited to academic anymore.” (Remark: Blackboard is a formal information system for in-class instruction in the university.)*

At the same time, their attitudes toward Facebook for academic learning varied:

*“I will enjoy [Facebook for academic learning] because Facebook provides a platform for group collaboration, encourages us to raise questions for discussion, makes us know more from one another and know more about the courses.”*

*“Sure! I check Facebook more often than email. Facebook is a ‘warmer’ place than Blackboard. I learn when giving comments to others and sharing my feelings with others.”*

*“Probably I will enjoy [Facebook for academic learning]. I will be more active and willing to learn, because it [Facebook] is also a tool for entertainment.”*

*“It [whether I will enjoy the academic learning on Facebook] depends on whether the usage of Facebook for education is interesting or not. I’ll learn if it can arise my interest.”*

*“I am not sure [whether I will enjoy academic learning on Facebook], since I am easy to get distracted and I cannot concentrate on the work I am doing.”*

*“I think it [Facebook] may have passive impact my studies, because I may not be concentrate on what I want to do.”*

The focus group discussions revealed both positive and negative aspects that Facebook might have on university students’ academic learning in university. The comments indicated that it might be not yet timely to apply OSN on academic learning but it is time to utilize Facebook to enrich students’ social life in university. Results from this theme discussion confirm to the findings by Paul et al. (2012) who suggested that students felt capable in OSN but were not willing to simply use OSN for academic learning. This pointed to the research appropriateness of social foci on the learning impact of OSN.

### Survey data collection

An anonymous online survey among university students was conducted to validate the above hypotheses. 187 valid individual responses were collected from a university. These respondents were young people with a mean age of 21.4 years, of which nearly half were male. On average, they logged onto Facebook four times per day and spent 1 h and a half on it per day. These young people established over 200 relationships on Facebook in which most were their university friends or previous high school friends. Table 1 shows the correlations of the related variables.

**TABLE 1:** Correlations of related variables.

Variables	1	2	3	4	5	6	7	8
1. OSN engagement	**0.816**							
2. Self-efficacy belief	0.247**	**0.860**						
3. Social acceptance	0.254**	0.423***	**0.748**					
4. Acculturation	0.218**	0.413**	0.530**	**0.756**				
5. Self-esteem	0.239**	0.654**	0.580**	0.481**	**0.825**			
6. Satisfaction	0.264**	0.596**	0.447**	0.480**	0.538**	**0.845**		
7. GPA	–0.022	0.314***	0.185**	0.116	0.228***	0.169**	–	
8. Gender	–0.044	0.138	0.003	–0.040	0.035	–0.017	–0.015	–
9. Study level	0.037	0.062	–0.087	–0.080	–0.030	0.134	0.103	–0.116

Correlation is significant at the 0.01 level: ***, at the 0.05 level: **, 2-tailed.

Values on the diagonal are square roots of AVE of constructs.

According to Podsakoff et al. (2003), precautions were taken to minimize the common method bias that could threaten the validity of the conclusions on the relationships between measures. First, multiple scales were used to measure these variables. Second, Harman’s single-factor method was used to further check for common method bias. No dominant factor emerging from the factor analyses was found, implying a low level of common method bias in this research design.

### Measures

Five- and seven-point Likert scales were used to measure the variables to reduce the possibility of common method bias (Podsakoff et al., 2003). Facebook was considered as the particular OSN context. Five items were adapted to measure for students’ OSN engagement (Ellison et al., 2007; Steinfield et al., 2008). Sampling questions such as “Facebook is part of my everyday activity,” “I feel I am part of the Facebook community,” “I would be sorry if Facebook shut shown” were asked in a 5-point Likert scale.

Self-efficacy, shaped by individuals’ perceived performance proficiency, was measured on four items in a 7-point Likert scale, which were adapted from Chao et al. (1994). Sampling questions were “I am confident about the adequacy of my academic skills and abilities,” “I feel competent conducting my course assignments,” and so on. Social acceptance was measured with five items, in which three were adapted from Morrison (2002) and two were adopted from Pascarella and Terenzini (1983). Sampling questions such as “The student friendships I have developed at my university have been personally satisfying,” “The students in the same cohort seem to accept me as one of them,” and “My interpersonal relationships with other students have had a positive influence on my personal growth, values and attitudes” were asked in a 5-point Likert scale. As for acculturation, three items related to the university culture were created, based on the interviews of the situated university students. It is common to generate measures for a specific culture. For example, “I am aware the value system of my university,” “I adapt myself to my university ’s culture” were asked in a 5-point Likert scale.”

Self-esteem was measured with five items which were developed by Rosenberg (1989), and further validated by Steinfield et al. (2008) in a university context. Sampling questions such as “I feel that I have a number of good qualities,” “I am able to do things as well as most other people,” “On the whole, I am satisfied with myself” and so on were asked in a 7-point Likert scale. Satisfaction with university life was measured with four items that were adapted from Rode et al. (2005). Sampling question such as “In most ways my life at my university is close to my ideal,” “The conditions of my life at my university are excellent” were asked in a 7-point Likert scale. Performance was measured by students’ self-reported cumulative GPA, ranging from 0 to 4. Several control variables were included, i.e., study level (from year 1 to 3) and gender (1 = male; 0 = female), as the inherent personal characters could affect students’ learning outcomes.

## Results and discussion

The analysis of the survey data was done in a holistic manner using the Partial Least Squares (PLS) with the bootstrap re-sampling procedure. Following the recommended two-stage analytical procedure (Anderson and Gerbing, 1988), the structural relationships were tested after the assessment of the measurement model.

### Measurement model assessment

The measurement model for reflective constructs was assessed by examining convergent validity and discriminant validity (Hulland, 1999). The convergent validity was assessed by examining composite reliability and average variance extracted (AVE) from the measures (Hair et al., 1998). As shown in Table 2, the composite reliability scores (ρ) of the reflective constructs exceed the threshold of 0.70, indicating that the measures are reliable (Nunnally, 1978). The AVE values range from 0.559 to 0.740, exceeding the recommended cut-off of 0.5. Further, all reflective items are significant on their path loadings at the 0.01 level (most above 0.70), providing evidence for convergent validity (Barclay et al., 1995).

**TABLE 2:** Assessment of convergent validity of constructs.

Constructs	Original Sample	Standard Error	T-value	Constructs	Original Sample	Standard Error	T-value
OSN engagement (ρ = 0.909; AVE = 0.666)	0.849	0.055	15.444	Social acceptance (ρ = 0.862; AVE = 0.559)	0.819	0.038	21.356
	0.773	0.060	12.897		0.839	0.034	24.954
	0.860	0.061	14.141		0.741	0.055	13.508
	0.826	0.041	19.98		0.661	0.077	8.586
	0.769	0.072	10.637		0.658	0.078	8.468
Self-efficacy belief (ρ = 0.919; AVE = 0.740)	0.884	0.023	39.268	Acculturation (ρ = 0.799; AVE = 0.571)	0.714	0.058	12.346
	0.905	0.017	54.691		0.736	0.050	14.792
	0.872	0.027	31.743		0.814	0.045	18.195
	0.775	0.061	12.647				
Self-esteem (ρ = 0.914; AVE = 0.681)	0.827	0.038	21.486	Satisfaction with Univ. life (ρ = 0.909; AVE = 0.714)	0.893	0.020	43.997
	0.862	0.026	33.753		0.892	0.017	51.07
	0.843	0.032	26.189		0.750	0.063	11.814
	0.821	0.030	27.006		0.838	0.029	28.737
	0.769	0.048	16.106				

Discriminant validity was tested by comparing the square roots of the AVE value of each construct to the correlation of the respective construct and other constructs. Table 1 also illustrates the discriminant validity statistics. The square roots of the AVE scores are all higher than the correlations among the constructs, demonstrating discriminant validity (Fornell, 1987).

### Structural model assessment

The results of full model testing (refer to Model 1 in Table 3), provide evidence for the impacts of OSN engagement on students’ social learning and the final learning outcomes. The coefficient of determination (R^2^) is typically used as a criterion of predictive power (Hair et al., 1998). The hypothesized model explains 56.8% variances of self-esteem, 47.7% variances of satisfaction with university life, and 11.6% variances of GPA-based performance. The standardized root mean square residual (SRMR) is a measure of approximate fit of the hypothesized model. The SRMR of the full model in Table 3 (Model 1) is 0.074, less than the recommended 0.08 (Hu and Bentler, 1999), indicating a good model fit.

**TABLE 3:** Independent mediation effect tests and comparisons.

Paths	Model 1 (Full model)		Model 2 (self-efficacy belief)		Model 3 (social acceptance)		Model 4 (acculturation)	
	β	t	β	t	β	t	β	t
OSN engagement → Self-esteem	0.231***	3.246	0.185***	2.918	0.151**	2.522	0.119***	2.914
OSN engagement → Satisfaction	0.211***	3.247	0.164***	2.754	0.125**	2.190	0.130***	3.048
OSN engagement → GPA	0.089**	2.165	0.086**	2.454	0.052*	1.685	0.030	1.267
OSN engagement → Self-efficacy belief	0.264***	2.948	0.270***	3.139				
OSN engagement → Social acceptance	0.246**	2.389			0.249***	2.717		
OSN engagement → Acculturation	0.239***	3.172					0.246***	3.598
Self-efficacy belief → Self esteem	0.483***	7.067	0.687***	14.126				
Self-efficacy belief → Satisfaction	0.418***	6.029	0.608***	9.215				
Self-efficacy belief → GPA	0.297***	3.730	0.318***	4.740				
Social acceptance → Self esteem	0.329***	4.024			0.608***	9.878		
Social acceptance → Satisfaction	0.165**	2.315			0.503***	6.674		
Social acceptance → GPA	0.102	0.940			0.207***	2.782		
Acculturation → Self-esteem	0.094	1.532					0.485***	8.709
Acculturation → Satisfaction	0.251***	3.453					0.528***	9.111
Acculturation → GPA	-0.063	0.621					0.122	1.588
*Control variables*								
*Gender → Self-esteem*	−*0.034*	*0.631*	−*0.071*	*1.267*	*0.036*	*0.583*	*0.055*	*0.871*
*Gender → Satisfaction*	−*0.048*	*0.877*	−*0.090*	*1.561*	*0.001*	*0.019*	*0.027*	*0.424*
*Gender → GPA*	−*0.049*	*0.667*	−*0.050*	*0.682*	−*0.002*	*0.028*	*0.003*	*0.032*
*Year → Self esteem*	−*0.036*	*0.727*	−*0.080*	*1.571*	*0.012*	*0.184*	*0.003*	*0.046*
*Year → Satisfaction*	*0.130* **	*2.435*	*0.087*	*1.432*	*0.163* ***	*2.635*	*0.168* ***	*2.867*
*Year → GPA*	*0.083*	*1.317*	*0.079*	*1.165*	*0.117*	*1.728*	*0.111*	*1.625*
**R^2^ for Self-esteem**	**56.8%**		**46.2%**		**37.0%**		**23.6%**	
**R^2^ for Satisfaction**	**47.7%**		**37.8%**		**26.8%**		**29.3%**	
**R^2^ for GPA**	**11.6%**		**10.9%**		**5.3%**		**2.5%**	

*p < 0.1, **p < 0.05, ***p < 0.01. The italic values represent the effects of control variables.

As hypothesized in H1_*a*∼*c*_, OSN can exert significant total effects on university students’ learning, leading to a higher level of self-esteem (β = 0.231, *t* = 3.246), satisfaction with university life (β = 0.211, *t* = 3.247), and GPA-based performance (β = 0.089, *t* = 2.165). OSN offers a new channel for the university students to learn in which they present themselves openly and share information with others. In this way, they foster psychological well-being and gain skills for academic or future career success.

The empirical results illustrate the mediation effects of the three attributions of social learning that perform different roles in transforming individuals’ OSN engagement into learning outcomes. First, university students’ OSN engagement significantly promotes their self-efficacy beliefs (β = 0.264, *t* = 2.948). Such beliefs substantially lead students to achieve the learning outcomes with the highest magnitudes of effects (β = 0.483, *t* = 7.707; β = 0.418, *t* = 6.029; β = 0.297, *t* = 3.730), compared with the other two attributions. Such results confirm the central role of self-regulation in social learning. The results strongly support H2 and H3_*a*∼*c*_.

Second, individual students’ OSN engagement is beneficial to their developing relationships with peers and gaining acceptance from peers (β = 0.246, *t* = 2.389), supporting H4. During the focus group discussions, the students commented that “*Facebook helps to establish and maintain my social network and friendship with others*,” and that “*Facebook encourages people to share feelings. I know more about friends as their activities and status are visible and traceable. I sometimes comment on their profiles. I also feel pleased when they comment on my profile.*” The social acceptance is significantly related to individuals’ self-esteem development (β = 0.329, *t* = 4.024) and satisfaction with life in university (β = 0.165, *t* = 2.315), but insignificantly related to their GPA (β = 0.102, *t* = 0.940).

Third, individuals’ social networking on Facebook is found to be significantly associated with their acculturation to the situated university (β = 0.239, *t* = 3.172), supporting H6. A learning university culture leads individuals to a higher level of satisfaction with life in the university (β = 0.251, *t* = 3.453), supporting H7_*b*_. In addition, the result indicates insignificant relationships between acculturation and self-esteem (β = 0.094, *t* = 1.532) and GPA (β = -0.063, *t* = 0.621).

Table 4 shows how self-efficacy belief, social acceptance and acculturation mediate the linkages between OSN engagement and learning outcomes, respectively. For self-esteem construction, students’ self-efficacy belief (β = 127, *t* = 2.574).and social acceptance (β = 0.081, *t* = 2.219) have the significant mediation effects. For students’ satisfaction with university life, their self-efficacy (β = 0.11, *t* = 2.577) and acculturation (β = 0.06, *t* = 2.364) exert the significant mediation effects. For students’ academic performance achievement, only their self-efficacy belief has the significant mediation effect (β = 0.079, *t* = 2.213). University students’ self-efficacy belief play the most important mediating role for transmitting OSN engagement into multifaceted learning outcomes.

**TABLE 4:** Mediation effect in the full model.

Mediation paths	Indirect effects	*t*-values	*P*-values
**OSN engagement → Self esteem**	**0.231**	**3.196**	**0.001**
OSN engagement → Self efficacy belief → Self esteem	0.127	2.574	0.01
OSN engagement → Social acceptance → Self esteem	0.081	2.219	0.027
OSN engagement → Acculturation → Self esteem	0.023	1.339	0.181
**OSN engagement** → **Satisfaction**	**0.211**	**3.168**	**0.002**
OSN engagement → Self efficacy belief → Satisfaction	0.11	2.577	0.01
OSN engagement → Social acceptance → Satisfaction	0.041	1.419	0.156
OSN engagement → Acculturation → Satisfaction	0.06	2.364	0.018
**OSN engagement** → **GPA**	**0.089**	**2.11**	**0.035**
OSN engagement → Self efficacy belief → GPA	0.079	2.213	0.027
OSN engagement → Social acceptance → GPA	0.025	0.845	0.398
OSN engagement → Acculturation → GPA	−0.015	0.568	0.571

Further, the independent mediation effects of self-efficacy, social acceptance and acculturation were checked by including only one of them separately (refer to Model 2∼4 in Table 3). The results of Model 2 and 3 demonstrate that individuals’ self-efficacy belief and social acceptance have appreciable independent mediation effects of linking OSN engagement to learning outcomes. The comparison between Model 1 and 3 indicates that the independent effect of social acceptance (e.g., between OSN engagement and GPA) is ruled out by individual efficacy beliefs, providing full support for H5_*a*_ and H5_*b*_ while partial support for H5_*c*_. The comparison between Model 1 and 4 implies that acculturation to a university environment always increases university students’ satisfaction with life at university. It also reveals that acculturation should have an impact on the development of self-esteem, offering partial support for H7_*a.*_ However, the relationship between acculturation and GPA remains insignificant in both models, thus providing no evidence to support H7_*c*_.

The results of the model tests and comparisons confirm the important role that the individual self plays in social learning. Although the independent mediation test illustrates that peers also largely influence individual students’ self-image formation and psychological well-being, the influence from peers could be diluted when individuals demonstrate self-efficacy beliefs. As an interaction between the students and the situated university, acculturation also has potential to improve the students’ positive recognition of themselves, but the magnitude of contribution is much less than that derived from the formation of self-efficacy and the obtaining of social acceptance. A learning university culture emotionally fosters students’ appreciation of their universities, however, the emotional commitment does not render skill improvement certain.

## Implications

### Theoretical implications

This study presents several implications for the extant literature, educational practices, and future research. The research model development on how OSN engagement affect learning accompanied by empirical validation contributes to the literature of online social networking for learning and the theories of social learning. This study shows multiple aspects of impacts that online social networking exerts on learning and reveal the mediating mechanisms *how* it influences individuals’ learning from a pedagogical perspective. The results enrich both social network and social learning research and extend applicability to OSN-based education.

The first contribution of this study lies in the illuminated positive impact of OSN on the social aspect of learning, adding values to the recent literature on OSN for education (e.g., Feyzi Behnagh and Yasrebi, 2020; Manca, 2020; Ouyang et al., 2021). The results demonstrate that OSN leads university students to “whole person” development with better psychological well-being and improved performance. OSN not only expands individuals’ large-scale networking capacity but also enables individuals to maintain close relationships with a small group of friends. It provides a new channel for students to fulfill the needs of self-expression, social comparison and information sharing. OSN allows university students to feel more comfortable in expressing themselves and interacting with peers and professors. It is noteworthy that OSN is only a portion of the students’ social life while their offline social interaction still plays a critical role for their achievement of desirable learning outcomes. However, the unique self-presentation and the largely expanded network scope via OSN engagement cannot be realized by the traditional networking channel.

The profile in OSN sites provides a new method for university students to model their images to the public. Regardless of whether the social networking profile functions as a copy, resemblance or simulacrum of oneself (Kreps, 2010), this study demonstrates that university students’ typing themselves into profiles facilitates a psychological process of self-conceptualization and recognition which is conducive to the formation of self-efficacy and self-esteem. Engagement in establishing various social relationships allows university students to learn more from knowledgeable others and learn more about the university, both of which are helpful for their moving from the broader edge of a community to the university center. The group profiling and visualization are important to trigger students to learn from the OSN learning environment and should be considered for e-learning application design (Troussas et al., 2015). By OSN, individual students can develop healthy cognition, emotions, and skills that are essential to the “whole person” development.

The second contribution lies in the revealed underlying mechanism, i.e., how the emergency of OSN can exert significant impacts on individuals’ learning outcomes. OSN has an influence not just on *what* students are learning but also on *how* they are learning. Evolving technologies are intricately woven into the fabric of social systems. Prior social network research primarily focuses on describing the structure or typology of a network (Granovetter, 1973; Coleman, 1990), while ignoring the potential transformation processes. This study explicitly proposes and empirically demonstrates that self-efficacy belief, social acceptance and acculturation link individuals’ OSN engagement to positive learning outcomes. These intervention effects are found at different levels of magnitude.

More specifically, the results underscore the central place that individuals’ efficacy beliefs take in the social learning process, in line with the student centric spirit of constructivism education (Piaget, 1953; Bandura, 1977, 1986; Lave and Wenger, 1991). OSN itself is a self-initiated and self-regulated learning process in which individuals establish relationships and seek for information with goal setting, and gradually obtain self-efficacy. The results also display the importance of peer interaction by bridging students’ OSN engagement to certain outcomes. Young people tend to observe others and compare themselves with others. They are indeed concerned about the feedback from peers. Most interpersonal interactions on social networking platforms are about feelings and making students feel comfortable with life. Students need a space to express their intended self, share pleasures, and release pressure. Moreover, it is worthy noting that university students’ acculturation via OSN increases their satisfaction with life at university. Thus, when self-regulation for social networking and social learning is emphasized, social acceptance and acculturation should also be highlighted for students as these social aspects help students articulate and transmit their role at universities.

### Practical implications

On a practical level, the social dimension of learning has always been of great significance to both individual learners and educational institutions. In the Internet era, the social behavior of human agency is changing. Most universities are experiencing socio-economic changes driven by state-of-the-art information technologies. From a university student standpoint, the findings show positive impacts of individuals’ OSN on their socialization processes in the situated university and ultimate learning outcomes. University students have become immersed in this emerging networking mode. But some students are consciously separating their time spent on social network sites from academic learning. As one commented, “*I could enjoy[learning on Facebook], but I like to keep Facebook and school separate*.” Such a distinct division between learning space and personal space may disruptively impact online social network learning usage.

Fortunately, the findings of this study help to mitigate this superficial gap between networking for leisure and networking for learning (Janković et al., 2016). Learning at university should be interpreted from the perspective of “whole person” development. University students’ OSN engagement, although it may originally be for fun, can help them establish efficacy belief, facilitate their observational learning from peers and help them to adapt to the university, and thus be beneficial for their self-esteem development, nurturing satisfaction with life, and even academic performance. Although this can support students in their “whole person” development for their future success, this does not mean that university students should spend more time on OSN platforms. Positive impact is assumed to be attained by those who have a high level of self-regulation capacity. Learning should be carefully designed so that it can address individual preferences to combine or separate social and personal space, as well as leisure and studying. E-learning applications that incorporate online social networking for education can be customized and developed as learning tools. The specific OSN-based learning tools have been shown be able to advance learners’ knowledge in computer science (Troussas et al., 2020, 2021).

As one coin has two sides, the possible negative impacts of intensively engaging in OSN platforms, such as distraction and addiction, should also be checked (Liu et al., 2017). During the focus group discussions, the interviewed students appeared to have different attitudes toward Facebook regarding academic learning. Some of them expressed positive attitudes, while others conveyed negative attitudes. For instance, students commented, “*I think it [Facebook] may have some passive impact on my studies, because I may not be able to concentration on what I want to learn.*” and “*I know some of my friends are addicted to Facebook too much, like in gaming. Somehow it will spend them a lot of time. I am worried about them. I sometimes will tell my best friends ‘Don’t do that’.*” The negative impacts even dark side of OSN (e.g., cyber-bullying) has been reported (Kift et al., 2010; Junco, 2012; Liu et al., 2017). These social problems are societal concerns and more research is definitely warranted. Thus, it is worthwhile to note that achieving desirable learning outcomes requires an appropriate social network configuration. OSN is only beneficial to those who choose to use it productively and appropriately.

Seen from the viewpoint of an educational institution, self-initiated and regulated learning could be good practice for university students, and part of such learning practice could be embedded into their social networking activities. The “self” plays an important role in one’s learning, since individuals set goals and regulate their activities for superior achievements. Thus, educational institutions should trust students and provide autonomy to students to learn individually and creatively. Self-regulated learning is not merely aimed at learning more knowledge but is aimed at endowing students with the capability, as well as the psychological confidence to learn independently, not only for their studies but also for the future. Learning autonomy is in line with the transformation of educational practices from the paradigm of teacher-centered learning to student-centered learning.

This study also illuminates that peers can greatly affect the social aspect of individuals’ learning. Peer interactions can partially promote self-initiated networking toward individuals’ psychological well-being development, such as in the formation of self-concept and self-esteem. Recognizing peer influence on various types of learning, previous research has recommended some educational practices, for example, peer mentoring (Sanchez et al., 2006) and peer-coaching (Parker et al., 2008). This study offers a complimentary approach for educational institutions to acknowledge peer influence, namely, providing a supportive infrastructure in which social networking activities can take place to increase interactions among students. From the “whole person” development perspective, students benefit from enriched extra-curriculum and social life.

Furthermore, educational practitioners can consider appropriately designing some generic educational practices on social networking sites, e.g., university orientation practices, university community of practices, and online mentoring practices. Such practices would allow students to form their own community, allow them to learn more about the university from the community, and therefore promote their university commitment and satisfaction. However, more research is warranted to explore the appropriateness of embedding social networking apps in curriculum design and exploring how to design it, as some of the interviewees showed negative attitudes regarding the usage of Facebook for academic learning. These students psychologically differentiate studying from learning which has a broader meaning.

## Limitations and future research

There are several limitations of this study that suggest the need for future research. First, Facebook was selected as the specific context. The students may also engage in other OSN sites. Although this treatment helps to reduce the complexity and variety of individuals’ OSN behavior that may also take place in many other OSN platforms, such specificity might make the investigation of OSN behavior inadequate. Future research can extend to investigate individuals’ OSN for learning in other platforms and the recently emerging live streaming platforms. Second, social learning theory interprets human behavior in terms of a continuous reciprocal interaction between cognitive, behavioral and environmental determinants. Although the research design of this study has data triangulation, it still limits the conclusion of causality as well as the detection of the interrelationship among the included factors. For example, the achieved learning outcomes can reciprocally lead individuals to more active engagement in OSN and more enthusiastic interactions with peers and the situations. A complete learning process is in a double loop rather than a single loop. Hence, future research can base on more rich objective data to gauge the reciprocity among social networking, socialization and learning outcomes, and adopt a longitudinal approach to track individuals’ behavior along a continuous time horizon. This would help to tease out the dynamism of individuals’ OSN behavior for learning.

## Data availability statement

The datasets presented in this study can be found in online repositories. The names of the repository/repositories and accession number(s) can be found below: Harvard Dataverse, doi: 10.7910/DVN/N9OG6Y.

## Ethics statement

Ethical review and approval was not required for the study on human participants in accordance with the local legislation and institutional requirements. Written informed consent from the participants was not required to participate in this study in accordance with the national legislation and the institutional requirements.

## Author contributions

YY contributed to the research design, data analysis, and manuscript writing.
